# Paediatric palliative care: recommendations for treatment of symptoms in the Netherlands

**DOI:** 10.1186/s12904-015-0054-7

**Published:** 2015-11-05

**Authors:** Rutger R. G. Knops, Leontien C. M. Kremer, A. A. Eduard Verhagen, on behalf of the Dutch Paediatric Palliative Care Guideline Group for Symptoms

**Affiliations:** Department of Paediatric Oncology, Emma Children’s Hospital/Academic Medical Centre, PO Box 22660, 1100 DD Amsterdam, The Netherlands; Department of Paediatrics, University Medical Centre Groningen/University of Groningen, Groningen, The Netherlands

**Keywords:** Palliative care, Child, Paediatrics, Symptoms

## Abstract

**Background:**

Children dying of a life threatening disease suffer a great deal at the end of life. Symptom control is often unsatisfactory, partly because many caregivers are simply not familiar with paediatric palliative care. To ensure that a child with a life-threatening condition receives high quality palliative care, clinical practice guidelines are needed. The aim of this study is to improve palliative care for children by making high quality care recommendations to recognize and relieve symptoms in paediatric palliative care.

**Methods:**

An extensive search was performed for guidelines and systematic reviews on paediatric palliative care up to year 2011. An expert panel combined the evidence with consensus to form recommendations on the treatment of symptoms in paediatric palliative care.

**Results:**

We appraised 21 guidelines and identified 693 potentially eligible articles of which four met our inclusion criteria. None gave recommendations on the treatment of symptoms in paediatric palliative care. Two textbooks and an adult palliative care website were eventually our main sources of evidence.

**Conclusion:**

Hardly any evidence is available for the treatment of symptoms in paediatric palliative care. By combining evidence for adult palliative care and the sparse evidence for paediatric palliative care with expert opinion we defined a unique set of high quality care recommendations to relieve symptoms and lessen the suffering of children in palliative care. These results are an important tool to educate caregivers on how to relieve symptoms in children in paediatric palliative care.

**Electronic supplementary material:**

The online version of this article (doi:10.1186/s12904-015-0054-7) contains supplementary material, which is available to authorized users.

## Background

Tens of thousands of children die each year in high income countries from trauma, prematurity, heritable disorders, and acquired illnesses. Even more children are coping with life-threatening conditions [1]. All these children need high quality palliative care. The American Academy of Paediatrics (AAP) has clearly stated that paediatric palliative care should be directed at the improvement of the quality of life of children dealing with a life-threatening condition and their families. Palliative care should be aimed at the prevention and relief of suffering by early identification and treatment of symptoms of physical, psychosocial, or spiritual nature and should be started at diagnosis and continued during the period of illness, irrespective of the outcome, either cure or death [2, 3]. All paediatricians, general physicians, and related professionals should become familiar with the provision of palliative care to children [3]. In paediatric palliative care greater attention should be given to symptom control and the overall wellbeing to lessen the suffering of children whose conditions make it unlikely that they will live into adulthood [4]. To ensure that children with a life-threatening condition receive high quality palliative care, clinical practice guidelines are needed. The aim of this study is to improve palliative care for children by making a systematic review with high quality care recommendations to recognize and relieve symptoms in paediatric palliative care.

## Methods

No written informed consent was needed for this study. The manual of the Dutch Evidence Based Guideline Development platform (EBRO platform) [5] was used for the methodology to develop a guideline, based on a systematic review with high quality care recommendations, for paediatric palliative care. After selection of topics, a step wise approach was followed to search in scientific literature for evidence in paediatric palliative care.

### Selection of topics

An expert panel consisting of different stakeholders in paediatric palliative care in the Netherlands was assembled. We asked the scientific associations of specialties that provide paediatric palliative care to select experts from different centres, whom we approached to participate in the expert panel. This expert panel was composed of 22 members and consisted of paediatric oncologists, paediatric neurologists, nurses, anaesthesiologists, psychologists, a hospice doctor, a palliative care specialist, a paediatric critical care specialist, a general practitioner, a physician for people with intellectual disabilities, health care managers, and patient/parent representatives. The expert panel was asked to create an inventory of the main symptoms during paediatric palliative care.

### Search for evidence

As a first step in our quest for evidence in paediatric palliative care we searched for guidelines in websites of international health care and guideline development organizations. The databases of Sumsearch (Medline, DARE, National Guideline Clearinghouse), Clinical Evidence of the BMJ group, Scottish Intercollegiate Guidelines Network (SIGN), and the Trip database were searched for paediatric palliative care guidelines up to year 2011. Selection of guidelines was based on title and carried out by two independent reviewers (M.U. and L.V.). The following inclusion criteria were used: 1) guideline directed at children (0 to 18 years of age) or adult guideline with separate recommendations for children, 2) guideline about palliative care (MESH-term). Palliative care guidelines for premature infants (gestational age less than 26 weeks) or resuscitation were excluded. The reason that palliative care for premature infants was excluded from this study, is that palliative care for this group of children takes place in a different setting (mainly neonatal intensive care units), with different symptoms and different symptom management. [6]. Resuscitation guidelines were excluded because they generally on unexpected, acute events, while the focus of our study was planned decision-making and palliative care in paediatric life threatening conditions. Members of the expert panel were also asked to supply additional guidelines.

As a second source for evidence we looked for systematic reviews on paediatric palliative care. The databases of Medline/Pubmed and The Cochrane Library were searched for systematic reviews on paediatric palliative care up to year 2011. The search strategy combined controlled vocabulary and text word terms for palliative care, children and systematic review. The search strategy is listed in Additional file 1: appendix 1. Data extraction of the included systematic reviews on paediatric palliative care was performed by two independent reviewers (M.U. and L.V.).

For the third and last step in our search for evidence in paediatric palliative care we used the sources most frequently used in daily practice in the Netherlands: two textbooks on paediatric palliative care (Wolfe [7], Goldman [8]) and the Pallialine website [9] (Dutch website with guidelines for adult palliative care).

To focus on high quality evidence that already has been integrated in guidelines and systematic reviews, we did not perform a systematic literature search for original studies in paediatric palliative care.

### Quality assessment

All retrieved guidelines were independently evaluated by two reviewers using the AGREE instrument [10]. Appraisal of guidelines by the AGREE-instrument can have as outcome that a guideline is recommended, not recommended or that recommendation is unclear. For this study we decided to include the recommended guidelines and the guidelines for which recommendation was unclear. By this measure we could also include guidelines based on multidisciplinary expert opinion in subjects where evidence is not available.

Quality assessment of the included systematic reviews on paediatric palliative care was performed by two independent reviewers and was based on the critical appraisal checklist of the CoCanCPG-project [11].

Quality assessment of the items that were derived from the textbooks [7, 8] and the Pallialine website [9] was done by two independent reviewers by checking the references. The level of evidence was based on the manual of the Dutch Evidence Based Guideline Development platform (EBRO-platform) [5] as shown in Table 1.

**Table 1:** Level of evidence for interventions

Level of evidence	Evidence is based on:
Level 1	Systematic review or at least two randomized clinical trials of good quality
Level 2	One randomized clinical trial or at least two case–control studies
Level 3	One case–control study or one cohort study
Level 4	Textbook or expert opinion

### Recommendations

The strength of a recommendation depends on the level of evidence and/or on general agreement [12]. The expert panel was asked to extend the evidence that resulted from our extensive search in literature with their expert opinion. The experts were able to reach consensus on all items. To combine the level of evidence with consensus we categorized the recommendations using the 3-colour scheme adapted from the American Heart Association [12].

Green is used for strong recommendations, based on high quality evidence (level 1 or 2) for children or for recommendations that can be extrapolated from high quality evidence (level 1 or 2) for adults, or for recommendations based on general agreement. The benefits of a green recommendation outweigh its disadvantages. Green recommendations should be performed.

Orange is used for an intermediate level of recommendations based on a moderate/weak level of evidence (level 3 or lower) for children or for recommendations that can be extrapolated from moderate/weak level of evidence (level 3 or lower) for adults, or for recommendations based on general agreement. The benefits are equal to or possibly outweigh the disadvantages. Orange recommendations should be considered.

Red is used for strong recommendations to avoid or prevent particular interventions. Red recommendations are based on high quality evidence (level 1 or 2) for children or for recommendations that can be extrapolated from high quality evidence (level 1 or 2) for adults, or for recommendations based on general agreement. Red recommendations have no benefits and possibly do harm. Red recommendations warn for interventions that should not be performed (Table 2). If evidence was conflicting and/or the expert panel could not reach consensus on the matter the term “controversy” was used. Controversial recommendations were omitted from the list of recommendations or were listed as recommendations to be considered.

**Table 2:** Levels of evidence and strength of recommendation

	Grading of Recommendation		
Level of evidence	Do	Consider	Don’t
Level 1	Strong Recommendation		Strong Recommendation
Level 2	Strong Recommendation		Strong Recommendation
Level 3		Moderate Recommendation	
Expert opinion	Strong Recommendation	Moderate Recommendation	Strong Recommendation

## Results

### Selection of topics

According to the expert panel inventory the main symptoms during paediatric palliative care that need to be addressed in a paediatric palliative care guideline are, in alphabetical order: 1) anxiety and depression, 2) bleeding and anaemia, 3) coughing and rattling, 4) dyspnoea, 5) fatigue, 6) nausea and vomiting, 7)neurological symptoms, 8) pain, and 9) pruritus.

### Search for evidence

The search for paediatric palliative guidelines in the websites of international health care and guideline development organizations identified a total of 21 potentially eligible guidelines. These 21 guidelines were appraised by the AGREE-instrument [10]. Eight guidelines were recommended after appraisal by the AGREE-instrument.

All of these guidelines gave recommendations about decision making in paediatric palliative care. But none of these recommended guidelines gave recommendations about recognizing and treating symptoms in paediatric palliative care.

The search for systematic reviews on paediatric palliative care in the databases of Medline/Pubmed and The Cochrane Library identified 693 potentially eligible articles. Screening the titles and abstracts of these articles excluded 673 articles. The remaining 20 articles were retrieved in full text for more detailed examination. Four [13–16] of the retrieved articles met all inclusion criteria and were used for data extraction. None of these systematic reviews gave recommendations about recognizing and treating symptoms in paediatric palliative care. The two textbooks on paediatric palliative care (Wolfe [7], Goldman [8]) and the Pallialine website [9] were eventually our main sources of evidence on recognizing and treating symptoms in paediatric palliative care. The evidence tables for the treatment of each symptom that was listed as main topic by our expert panel are depicted in Additional file 1: Appendix 3.

### Recommendations

The recommendations, based on evidence and/or consensus, for the treatment of symptoms of each topic are listed in Table 3.

**Table 3:** Recommendations for treatment of symptoms in paediatric palliative care

*- Anxiety and depression*	
Do	• Consult a psychologist, paediatric psychiatrist, if necessary a physician for people with intellectual disabilities or someone of a similar discipline.
	• Decide in deliberation with the parents the mode of treatment for the anxiety and/or depression of the child.
	• Involve a spiritual caregiver (possibly of the family’s own conviction) to help with existential philosophical questions.
	• Offer relaxation and distraction techniques in case of anxiety.
Consider	• Consider selective serotonin reuptake inhibitors (SSRI’s) in case of anxiety, whether or not accompanied by depression.
	• Consider methylphenidate in case of depression.
	• Consider the help of experts for self-hypnosis.
*- Bleeding and anaemia*	
Do	• In case of mild bleeding and shortage of coagulation factors give desmopressin, tranexamic acid and/or vitamin K.
	• In case of severe bleeding give fresh frozen plasma and/or recombinant factor VII.
	• In case of a life threatening bleeding and DNR-order explain symptoms and treat dyspnoea with morphine, diazepam or midazolam.
	• In case of thrombosis give low molecular weight heparin.
Consider	• Consider blood transfusion in anaemia (Hb<5,0 mmol/L).
	• Consider desmopressin for bleeding caused by mild thrombocytopenia.
	• Consider for nose bleeds local adrenalin, xylometazolin, spongostan or local coagulation by an ENT-physician.
	• Consider in case of bleeding platelet transfusions.
	• Consider transfusions of platelets before physical activities.
	• Consider heparin in case of thrombosis.
Don’t	• Do not give vitamins and/or nutritional supplements.
	• Do not give erythropoietin.
*- Coughing and rattling*	
Do	*Coughing*
	• Give counselling and do breathing exercises.
	• Give morphine if coughing leads to discomfort.
Consider	*Coughing*
	• Consider codeine, dextromethorphan or noscapin.
	• Consider physical therapy for productive coughing.
	• Consider “huffing” (breathing technique in which expiration is performed with open glottis).
	• Consider expirational compression of thorax.
	• Consider different postures (coughing while standing or sitting is more effective).
	• Consider inhalation of physiological saline (NaCl 0.9 %).
	*Rattling*
	• Consider suction.
	• Consider lying on one’s side.
	• Consider anti-cholinergic medication.
*- Dyspnoea*	
Do	• Give information and do breathing exercises
	• Create a calm and quiet atmosphere
	• Give morphine if dyspnoea leads to discomfort
Consider	• Consider referral to physical therapist for “Neuro electrical muscle stimulation and chest wall vibration”.
	• Consider the use of a fan to cool the face.
	• Consider the use of experts for self-hypnosis.
	• Consider the use of lorazepam or midazolam, in combination with morphine to lessen the experienced discomfort, especially in case of anxiety.
	• Consider inhalation of physiological saline (NaCl 0.9 %) or hypertonic saline (NaCl 3 %) in case of thick mucus.
	• Consider the inhalation of corticosteroids, airway dilators and mucolytic drugs in case of bronchial obstruction and/or asthma.
	• Consider the supply of oxygen.
Don’t	• Do not nebulize morphine.
*- Fatigue*	
Do	• Treat electrolyte disorders, dehydration and/or malnourishment.
	• Treat comorbidity like asthma, infection, pain and/or pruritus.
	• Discuss in case of depression counselling and antidepressants.
	• Encourage a regular sleep-wake rhythm and avoid stimulants.
	• Give advices for relaxation and distraction.
	• Give psycho education for managing fatigue.
	• Emphasize the importance of the balance between physical activities and rest.
	• Focus on what the child can do and not on what he/she is unable to do anymore.
	• Keep a diary to get insight in fatigue and activities.
	• Advise fatigue reducing activities.
	• Advise a regular sleeping scheme.
	• Consult a physical therapist for an exercise program.
Consider	• Consider corticosteroids.
	• Consider melatonin for sleeping disorders.
	• Consider blood transfusion if haemoglobin drops below 5 mmol/l.
	• Consider short lasting treatment with benzodiazepines for sleeping disorders.
	• Consider stopping medication which might have fatigue as side effect.
	• Consider the treatment of other underlying symptoms.
	• Consider stimulating bedridden children to get out of bed regularly.
	• Consider consulting a psychologist for psychotherapy or support.
	• If all of the above does not help, consider methylphenidate.
*- Nausea and vomiting*	
Do	• Distract the child, especially in case of anxiety.
	• Combine non-medical treatment with medical treatment.
Consider	• Consider dietary advice.
	• Consider consultation of a physical therapist, psychologist and/or music therapist.
	• Consider hypnosis.
	• Consider a step-wise approach in medical treatment:
	Step 1:
	• 5-HT3-receptor antagonist and/or
	• D2-receptor antagonist
	• H1-, and ACh-receptor antagonists
	Step 2:
	• Corticosteroids
	• Low dose benzodiazepine
	• Replace a step 1 drug with another member of the same family of drugs (rotation).
	• Replace phenothiazine by as well an antihistamine as a dopamine receptor antagonist.
	Step 3:
	• Aprepitant
	• Cannabis
	• Low dose propofol
*- Neurological symptoms*	
Do	*Seizures*
	• Give midazolam or diazepam in case of seizures lasting over 5 min or in case of several short seizures.
	• Consult a paediatric neurologist.
	*Diplopia*
	• Give an eye patch.
	*Non-closure of (one of) the eyes*
	• Give during the day methylcellulose eye drops.
	• Give at night oculentem simplex eye ointment.
	*Swallowing disorders*
	• Try to prevent aspiration.
	• Give optimal feeding fitting the stage of disease.
	• Offer fluids with a straw.
	• Make sure there are pauses between swallowing.
	• Let the child sit straight.
Consider	*Seizures*
	• Consider maintenance treatment, like clonazepam, levetiracetam, valproic acid, carbamazepine, phenobarbital, clobazam, phenytoin or midazolam.
	*Non-seizure movements*
	• Consider consulting a paediatric neurologist.
	• Consider bipiridene to treat acute dystonia caused by anti-emetics.
	• Consider baclofen or benzodiazepines to treat unpleasant movements.
	• Consider injecting botulinum toxins for treatment of local spasticity.
	*Swallowing disorders*
	• Consider a nasogastric tube to prevent aspiration.
	• Consider a nasogastric tube to guarantee intake.
	• Consider thickening of the food.
*- Pain*	
Do	• Treat pain according to a set (time) scheme, use the most suitable way and adjust to the needs of the child.
Consider	• Consider melatonin for headaches and sleeping disorders.
	• Consider complementary therapies.
*- Pruritus*	
Do	• Prevent intertrigo.
	• Treat crusting with damp towels.
	• Give ointments for dry skin.
	• Give ointments with anti-pruritus supplements (menthol).
	• Give ointments with corticosteroids for eczema.
	• Give disinfectants, anti-fungal and/or antibiotic ointments for infected skin.
	• In case of opioid-associated pruritus give opioid antagonists or rotate opioids.
Consider	• Consider cooling the skin.
	• Consider hypnosis.
	• Consider antihistamines.
	• Consider serotonin antagonists.
	• Consider H2-receptor antagonists.
	• Consider additional chemotherapy and/or steroids for lymphoma-associated pruritus.
	• In case of cholestasis-associated pruritus consider stenting, H2-receptor antagonists, mirtazapine, serotonin antagonists, rifampicin, phenobarbital, cholestyramine, ursodeoxylcholic acid or prednisone (if there are no other solutions).

## Discussion

Hardly any evidence is available for the treatment of symptoms in paediatric palliative care. By maintaining a systematic approach we were able to define a unique set of high quality care recommendations to relieve symptoms and lessen the suffering of children in palliative care. Although care for children differs immensely from adult care, literature on adult palliative care is the main source from which recommendations for paediatric palliative care can be deduced from. By combining evidence for adult palliative care and the sparse evidence for paediatric palliative care with paediatric expert opinion we defined recommendations for treating symptoms in paediatric palliative care.

Paediatric palliative care is complex. The management of the physical, psychosocial, and spiritual needs of a child with a life threatening condition is intense and difficult. Palliative care for children is, in comparison with adults, also relatively rare and views on how to handle and cope with dying children differ in many cultures. This complexity and rarity requires a specialized multidisciplinary approach to deliver optimal care [17]. Caregivers dealing with a child in palliative care should be able to consult paediatric palliative care teams, especially in case of pain management. As paediatric palliative care teams are not widely or immediately available, caregivers dealing with a child in palliative care need to know how to manage symptoms [18]. Children dying of a life threatening disease suffer a great deal at the end of life. Symptom control in children dying of cancer is often unsatisfactory at this stage of disease [4]. Symptom control could benefit from greater awareness, prevention, and early recognition of symptoms by caregivers in paediatric palliative care. Better treatment of symptoms is also necessary to ease the suffering of children with life-threatening conditions. The results of this study are an important tool to educate caregivers who are inexperienced in palliative care for children. These recommendations can be used as a guideline and will help to make decisions on the treatment of symptoms in children with life-threatening conditions in high income countries.

In the last decade many standards, guidelines and quality improvement strategies have been developed for adult palliative care. This has been a major contribution to the integration of palliative care into daily practice and has improved the quality of adult palliative care [19]. Because of its complexity and rarity, paediatric palliative care has seen a much slower progress. Local initiatives have tried to give guidance in paediatric palliative care, but standardization of care is lacking. By using an expert panel consisting of many members with different professional backgrounds, we provided broad multidisciplinary support on a national level for the standardization of the recommendations on the treatment of symptoms in paediatric palliative care.

The recommendations on the treatment of symptoms in paediatric palliative care were categorized according to a colour scheme: green for “do”, orange for “consider” and red for “don’t”. Evidence for green and red recommendations is of a high enough level that no further discussion is needed whether these recommendations should or respectively should not be performed. Attention should be called to the orange recommendations. As the effectiveness of orange recommendations is unclear, shared decision making is needed to decide whether these recommendations should be performed or not. Future research should be directed at the questions raised by the orange recommendations and should clarify whether they should be recommended or should be avoided.

This study will be helpful in the international debate on improving the care for children with life-threatening conditions. Palliative care is a wide concept which also encompasses end-of-life care. Decision making in the last stage of life is difficult and is complicated by delicate ethical, legal, religious, and political issues. In the Netherlands, decisions to actively end a patient’s life because of hopeless and unbearable suffering have been formalized in laws and other regulations, both for adults and for new-borns [20, 21]. It seemed odd that this specific part of end-of-life care received so much attention, while there were no formal guidelines available for the palliative care for children in the Netherlands. Our approach can be seen as an important step towards closing the gap between treatment of suffering in children in the context of palliative care and end-of-life care.

A limitation of this study is the lack of evidence in symptom control in paediatric palliative care, which also indicates the lack of our knowledge in general on this matter. To keep a broad outlook on paediatric palliative care and to focus on high quality evidence that already has been integrated in guidelines and systematic reviews, we did not perform a systematic literature search for original studies in paediatric palliative care. This implies that evidence from studies which have not (yet) been included in systematic reviews and guidelines could have been missed.

As mentioned above the main source of evidence for recommendations for paediatric palliative care was adult palliative care literature. As literature on adult palliative care is predominantly oriented towards cancer, the recommendations in this study focus also on symptoms experienced in cancer. The range of symptoms experienced in paediatric palliative care differ however in cancer and non-cancer patients [22]. Taken into account that the majority of children in palliative care in North America have non-cancer diagnoses [23, 24] it should be stated that this study does not cover the full range of symptoms that may be encountered in paediatric palliative care.

The list of recommendations for the treatment of symptoms in paediatric palliative care was used to develop the Dutch guideline for paediatric palliative care, which also addresses decision making and the organisation of paediatric palliative care in the Netherlands [25]. This guideline was approved by a broad selection of scientific associations who all participate in the palliative care for children in the Netherlands (Additional file 1: Appendix 2). As mentioned above, the search for for paediatric palliative care guidelines was performed in the year 2011. Paediatric palliative care guidelines published after 2011 will be included after discussion in the revision and extension of the Dutch guideline for paediatric palliative care [26, 27].

Although the importance of good palliative care receives a growing amount of interest, the focus of research has been mainly on the organization of palliative care and not on the management of symptoms. As mentioned above, hardly any evidence is available on symptom control in paediatric palliative care. Randomized controlled trials in palliative care are complicated because of many methodological difficulties and ethical issues [28]. Because of these issues randomized trials are often considered inappropriate or even not possible in palliative care. Well-designed observational studies can provide valuable information in palliative care [29]. An enormous task awaits researchers to fill out the gaps in this field.

## Conclusions

In conclusion, by combining evidence for adult palliative care and the sparse evidence for paediatric palliative care with paediatric expert opinion we were able to define an extensive list of recommendations for the treatment of symptoms in paediatric palliative care. Paediatric palliative care should be directed at the improvement of the quality of life of children dealing with a life-threatening condition and their families. The results of this study can be used as a guideline and are an important tool to educate caregivers on how to relieve symptoms in children with life threatening conditions and improve quality of paediatric palliative care in high income countries.

## Additional file

Additional file 1: Appendix 1: Search strategy. Appendix 2: List of scientific associations. Appendix 3: Evidence table for the treatment of symptoms, References of appendix 3. (DOC 201 kb)
