# Supplementary material for: Paediatric palliative care: recommendations for treatment of symptoms in the Netherlands
Source: BMC Palliat Care. 2015 Nov 5;14:57. doi: 10.1186/s12904-015-0054-7 (PMC4634793; doi:10.1186/s12904-015-0054-7)
Supplement: Additional file 1: — Appendix 1: Search strategy. Appendix 2: List of scientific associations. Appendix 3: Evidence table for the treatment of symptoms, References of appendix 3. (DOC 201 kb) [file 12904_2015_54_MOESM1_ESM.doc]

**Additional file 1**

**Appendix 1** Search strategy

Palliative care OR Care, Palliative OR Palliative Treatment OR Palliative Treatments OR Treatment, Palliative OR Treatments, Palliative OR Therapy, Palliative OR Palliative Therapy OR Palliative Medicine OR Medicine, Palliative OR Palliative Surgery OR Surgery, Palliative OR pediatric palliative care OR special health care OR children's hospice OR Hospice care OR Terminal care

Infant OR infan* OR newborn OR newborn* OR new-born* OR baby OR baby* OR babies OR neonat* OR perinat* OR postnat* OR child OR child* OR schoolchild* OR schoolchild OR school child OR school child* OR kid OR kids OR toddler* OR adolescent OR adoles* OR teen* OR boy* OR girl* OR minors OR minors* OR underag* OR under ag* OR juvenil* OR youth* OR kindergar* OR puberty OR puber* OR pubescen* OR prepubescen* OR prepuberty* OR pediatrics OR pediatric* OR paediatric* OR peadiatric* OR schools OR nursery school* OR preschool* OR pre school* OR primary school* OR secondary school* OR elementary school* OR elementary school OR high school* OR highschool* OR school age OR schoolage OR school age* OR schoolage* OR infancy OR schools, nursery OR infant, newborn

((systematic review[tiab] OR review literature[mh] OR review[tiab] OR reviews[tiab] OR review[pt] OR systematic literature review[tiab] OR systematic literature review[tiab]) OR (meta analysis[pt] OR meta analysis[mh] OR meta analysis[tiab] OR metaanalysis[tiab] OR meta analyses[tiab]) OR (guidelin*[tiab] OR guideline[pt] OR evidence[tiab]) OR (Search AND "Cochrane Database Syst Rev"[Journal])) AND ((databases[mh] OR bibliography[tiab]) OR ((medline[tiab] OR embase[tiab] OR pubmed[tiab] OR cinahl[tiab] OR cochrane[tiab]) OR medline[mh] OR pubmed[mh]) OR (search*[tiab] OR handsearch*[tiab]))

**Appendix 2** List of scientific associations

- Royal Dutch Pharmaceutical Society (KNMP)

- Dutch College of General Practitioners (NHG)

- Dutch Association of Psychologists (NIP)

- Dutch Association of Anaesthesiologists (VNA)

- The Netherlands’ Society of Physicians for People with Intellectual Disabilities

(NVAVG)

- Dutch Association for Bio-Ethics (NVBE)

- Dutch Association of Paediatric Neurologists (NVKN)

- Dutch Childhood Oncology Group (DCOG)

- PAL Foundation (Stichting PAL)

- Association for Adults, Children and Metabolic Disorders (VKS)

- Dutch Association for Nurses and Caregivers (V&VN)

- Association for Parents, Children and Cancer (VOKK)

- Comprehensive Cancer Centre the Netherlands (CCCN)

- Paediatric Association of the Netherlands (NVK)

**Appendix 3** Evidence table for the treatment of symptoms

| **Treatment** | **Level of evidence**  **for children** | **Level of evidence**  **for adults** | **Effectiveness** |
| --- | --- | --- | --- |
| ***Anxiety and depression*** |  |  |  |
| General interventions for anxiety (cognitive and behavioural interventions) | Level 3 [1-4] | Level 1 [5,6] | Effective (adult)  Possibly effective (child)* |
| Benzodiazepines  for anxiety | Level 4 [7,8] | Level 3 [9-11] | Possibly effective (adult)  Possibly effective (child) |
| SSRI’s  for anxiety | Level 4 [12] | Level 1 [13] | Effective (adult)  Possibly effective (child) |
| SSRI’s for anxiety and depression for children with cancer | Level 4 [14-16] | Level 1 [13] | Effective (adult)  Possibly effective (child) |
| General interventions for depression (cognitive and behavioural interventions) | Level 1 [7] | Level 1 [17-20] | Effective (adult)  Effective (child) |
| SSRI’s for depression | Level 3 [12,21,22] | Level 1  [18-20,23,24] | Effective (adult)  Possibly effective (child) |
| Tricyclic antidepressants | Controversy [25,26] | Level 1 [18-20,24] | Effective (adult)  Controversy (child) |
| Methylphenidate | Level 4 [27] | Level 3 [28,29] | Possibly effective (adult)  Possibly effective (child) |

| **Treatment** | **Level of evidence**  **for children** | **Level of evidence**  **for adults** | **Effectiveness** |
| --- | --- | --- | --- |
| ***Dyspnoea*** |  |  |  |
| Neuro electrical muscle stimulation and chest wall vibration |  | Level 1 [30] | Effective* |
| Counselling and breathing exercises |  | Level 2 [30-36] | Effective |
| Acupuncture |  | Controversy [30] | Controversy |
| Cooling |  | Level 4 [30,37-39] | Possibly effective |
| Self-hypnosis | Level 4 [40] |  | Possibly effective |
| Quiet environment | Level 4 [7] |  | Possibly effective* |
| Inhalation of physiological or hypertonic saline |  | Level 4 [41] | Possibly effective |
| Morphine (oral, iv) |  | Level 1 [31,35,37,42-44] | Effective |
| Morphine (inhalation) |  | Level 1 [45-46] | Not effective |
| Benzodiazepines  for dyspnoea with anxiety |  | Level 2 [47] | Possibly effective |
| Corticosteroids |  | Level 4 [44,48] | Possibly effective |
| Airway dilators |  | Level 4 [48] | Possibly effective |
| Oxygen |  | Controversy [31,35,49-52] | Controversy |

* = recommendation based on general agreement

| **Treatment** | **Level of evidence**  **for children** | **Level of evidence**  **for adults** | **Effectiveness** |
| --- | --- | --- | --- |
| ***Bleeding and anaemia*** |  |  |  |
| Blood transfusion | Level 3  [7,12,53-54] | Level 3 [55] | Possibly effective (adult)  Possibly effective (child) |
| Vitamins | Level 4 [7] |  | Not effective |
| Iron | Level 4 [7] |  | Not effective |
| Erythropoietin  (Dependent on condition) | Level 2 [56,57] | Level 2 [58] | Not effective (adult)  Not effective (child) |
| Desmopressin | Level 4 [7] |  | Possibly effective |
| Vitamin K | Level 4 [7] |  | Possibly effective |
| Recombinant Factor VII | Level 4 [7] |  | Possibly effective |
| Tranexamenic acid | Level 4 [7,12,59,60] |  | Possibly effective |
| Adrenalin | Level 4 [7] |  | Possibly effective |
| Xylometazolin | Level 4 [7] |  | Possibly effective |
| Platelet transfusion | Level 4 [7,61] |  | Possibly effective |
| Fresh frozen plasma | Level 4 [7] |  | Possibly effective |
| Heparin | Level 4 [12] |  | Effective |
| Low molecular weight heparin | Level 4 [12] | Level 1 [62-64] | Effective |

| **Treatment** | **Level of evidence**  **for children** | **Level of evidence**  **for adults** | **Effectiveness** |
| --- | --- | --- | --- |
| ***Coughing*** |  |  |  |
| Counselling, physical therapy, “huffing”, inhalation physiological saline |  | Level 4 [13] | Possibly effective |
| Dextromethorphan | Level 4 [7] | Level 3 [65-67] | Possibly effective (adult)  Possibly effective (child) |
| Codeine and other opioids | Level 4 [7] | Level 3 [65-69] | Possibly effective (adult)  Possibly effective (child) |
| ***Rattling*** |  |  |  |
| Suction | Level 4 [7] | Level 4 [13] | Not effective (adult)  Effective (child) |
| Anticholinergic medication | Level 4 [7] | Level 3 [70-76] | Possibly effective (adult)  Possibly effective (child) |

| **Treatment** | **Level of evidence**  **for children** | **Level of evidence**  **for adults** | **Effectiveness** |
| --- | --- | --- | --- |
| ***Pruritus*** |  |  |  |
| Cooling, normal skin care | Level 4 [7] | Level 1 [77,78] | Effective (adult)  Possibly effective (child) |
| Hypnosis |  | Level 3 [79-81] | Possibly effective |
| Antihistamines | Level 3 [82] |  | Possibly effective |
| Naloxone  (opioid-associated pruritus) | Level 1 [83] | Level 1 [84] | Effective |
| Ondansetron  (opioid-associated pruritus) |  | Level 1 [85-89] | Effective* |
| Cimetidine |  | Level 4 [90-96] | Possibly effective |
| Prednisone and cimetidine |  | Level 4 [97] | Possibly effective |
| SSRI | Level 3 [98] | Level 2 [99] | Possibly effective (adult)  Possibly effective (child) |
| Ondansetron  (cholestasis associated pruritus) |  | Level 2 [100,101] | Effective* |
| Mirtazapine (cholestasis-associated pruritus) |  | Level 4 [102-104] | Effective |
| SSRI (cholestasis-associated pruritus) |  | Level 3 [105-109] | Effective |
| Cholestyramine (cholestasis-associated pruritus) |  | Level 3 [110-112] | Effective |
| Ursodeoxycholic acid (cholestasis-associated pruritus) | Level 3 [113,114] |  | Effective |
| Rifampicin (cholestasis-associated pruritus) | Level 3 [115] | Level 1 [112,116] | Effective (adult)  Effective (child) |
| Phenobarbital (cholestasis-associated pruritus) | Level 3 [117-118] |  | Possibly effective |
| Naloxone (cholestasis-associated pruritus) | Level 3 [119] | Level 1  [112,120-123] | Effective (adult)  Effective (child) |

* = recommendation based on general agreement

| **Treatment** | **Level of evidence**  **for children** | **Level of evidence**  **for adults** | **Effectiveness** |
| --- | --- | --- | --- |
| ***Nausea and vomiting*** |  |  |  |
| Dietary advices |  | Level 4 [124] | Possibly effective |
| Hypnosis | Level 2 [125-129] |  | Effective* |
| Massage |  | Level 2 [130-132] | Possibly effective |
| Distraction | Level 3 [125] | Level 3 [133] | Possibly effective (adult)  Effective (child) |
| Psychological intervention |  | Level 1 [134,135] | Effective* |
| Music therapy |  | Level 3 [136] | Possibly effective |
| Acupuncture | Controversy [137] | Controversy  [138-143] | Controversy |
| 5-HT2-receptor antagonist (chemo induced nausea) | Level 2 [144-146] | Level 1  [139,147-152] | Effective (child)  Effective (adult) |
| 5-HT2-receptor antagonist (other causes nausea) |  | Level 2 [153-161] | Effective |
| Metoclopramide | Level 4 [7] | Level 4 [7] | Effective |
| Haloperidol | Level 4 [7] | Level 4 [162,163] | Effective (child)  Possibly effective (adult) |
| Domperidon | Level 4 [7] | Level 4 [7] | Effective (child) |
| Cyclizine | Level 4 [7] | Level 4 [13] | Effective (child)  Possibly effective (adult) |
| Promethazine | Level 4 [7] |  | Effective (child) |
| (Butyl)scopolamine | Level 4 [7] | Level 4 [13] | Effective (child)  Possibly effective (adult) |
| Chlorpromazine | Level 4 [7] |  | Effective (child) |
| Levomepromazine | Level 4 [7] | Level 3 [164-166] | Effective (child)  Effective (adult) |
| Aprepitant (chemo induced nausea) | Level 3 [167,168] | Level 1  [139,147-149,168] | Effective (child)  Effective (adult) |
| Aprepitant (other causes nausea) | Level 3 [169] |  | Effective (child) |
| Cannabis |  | Level 3 [170] | Effective (adult) |
| Dexamethasone (combined with other anti-emetics) |  | Level 3 [171-174] | Possibly effective (adult) |
| Benzodiazepines (nausea after surgery) | Level 2 [175] | Level 1 [176] | Effective (child)*  Possibly effective (adult) |
| Benzodiazepines (chemo induced nausea) | Level 2 [177,178] |  | Effective (child)* |
| Propofol (chemo induced nausea) | Level 4 [7] | Level 3 [179,180] | Possibly effective (child)  Effective (adult) |
| Propofol  (nausea after surgery) | Level 4 [7] | Level 1 [181,182] | Possibly effective (child)  Effective (adult) |

* = recommendation based on general agreement

| **Treatment** | **Level of evidence**  **for children** | **Level of evidence**  **for adults** | **Effectiveness** |
| --- | --- | --- | --- |
| ***Pain*** |  |  |  |
| Integrative therapies | Level 2 [183,184] |  | Possibly effective* |
| Psychological therapy | Level 1 [185] |  | Effective |
| Acetaminophen | n.a. [186] |  | Effective |
| NSAID | n.a. [186] |  | Effective |
| Tramadol | n.a. [186] |  | Possibly effective |
| Codeine | n.a. [186] |  | Controversy |
| Morphine | n.a. [186] | Level 3 [187] | Effective |
| Oxycodone | n.a. [186] |  | Possibly effective |
| Buprenorphine | n.a. [186] |  | Possibly effective |
| Corticosteroids | n.a. [186] |  | Possibly effective |
| Amitriptyline |  | Level 1 [188,189] | Effective (adult) |
| Gabapentin  Pregalabine |  | Level 1 [189-191] | Effective (adult) |
| Phenytoin  Carbamazepine  Valproate acid |  | Controversy [189] | Controversy |
| Opioids |  | Level 1 [189,192] | Effective |

n.a. = not applicable * = recommendation based on general agreement

| **Treatment** | **Level of evidence**  **for children** | **Level of evidence**  **for adults** | **Effectiveness** |
| --- | --- | --- | --- |
| ***Fatigue*** |  |  |  |
| Blood transfusion for anemia | Level 4 [7] | Level 3 [193] | Possibly effective (child)  Effective (adult) |
| Psycho education | Level 2 [194,195] |  | Effective (child) |
| Sleep hygiene | Level 4 [7,96] | Level [13] | Possibly effective (child)  Possibly effective (adult) |
| Exercise | Level 4 [197] | Level 3 [198-204] | Possibly effective (child)  Effective (adult) |
| Nutrition | Level 4 [196] | Level 4 [13] | Effective (child)  Effective (adult) |
| Psychotherapy and counselling | Level 4 [7] | Level 1 [205-208] | Possibly effective (child)  Effective (adult) |
| Psych stimulants/  Methylphenidate |  | Controversy  [209-212] | Controversy |

**References of appendix 3**

1. Spirito A, Kazak AE. Effective and emerging treatments in pediatric psychology.Oxford University Press. Oxford,UK. 2006.

2. Powers SW. Empirically supported treatments in pediatric psychology: procedure-related pain. *J Pediatr Psychol* 1999;24(2):131-145.

3. Barrera ME, Rykov MH, Doyle SL. The effects of interactive music therapy on hospitalized children with cancer: a pilot study. *Psychooncology* 2002;11(5):379-388.

4. Robb SL, Ebberts AG. Songwriting and digital video production interventions for pediatric patients undergoing bone marrow transplantation, part I: an analysis of depression and anxiety levels according to phase of treatment. *J Pediatr Oncol Nurs* 2003;20(1):2-15.

5. Sheard T, Maguire P. The effect of psychological interventions on anxiety and depression in cancer patients: results of two meta-analyses. *Br J Cancer* 1999;80(11):1770-1780.

6. Uitterhoeve RJ, Vernooy M, Litjens M et al. Psychosocial interventions for patients with advanced cancer - a systematic review of the literature. *Br J Cancer* 2004;91(6):1050-1062.

7. Wolfe J, Hinds PS, Sourkes BM. Textbook of Interdisciplinary Pediatric Palliative Care. Elsevier. Philadelphia,PA. 2011.

8. Manassis K. Childhood anxiety disorders: lessons from the literature. *Can J Psychiatry* 2000;45(8):724-730.

9. Henderson M, MacGregor E, Sykes N, Hotopf M. The use of benzodiazepines in palliative care. *Palliat Med* 2006;20(4):407-412.

10. Jackson KC, Lipman AG. Drug therapy for anxiety in palliative care. *Cochrane Database Syst Rev* 2004;1:CD004596.

11. Stiel S, Krumm N, Schroers O, Radbruch L, Elsner F. [Indications and use of benzodiazepines in a palliative care unit]. *Schmerz* 2008;22(6):665-671.

12. Goldman A, Hain R, Liben S. Oxford Textbook of Palliative Care for Children. Oxford University Press. Oxford,UK. 2006.

13. Comprehensive Cancer Center the Netherlands. Guidelines palliative care. http://www.pallialine.nl/. 2013.

14. Axelson DA, Birmaher B. Relation between anxiety and depressive disorders in childhood and adolescence. *Depress Anxiety* 2001;14(2):67-78.

15. Gurley D, Cohen P, Pine DS, Brook J. Discriminating depression and anxiety in youth: a role for diagnostic criteria. *J Affect Disord* 1996;39(3):191-200.

16. Collins JJ, Byrnes ME, Dunkel IJ et al. The measurement of symptoms in children with cancer. *J Pain Symptom Manage* 2000;19(5):363-377.

17. Akechi T, Okuyama T, Onishi J, Morita T, Furukawa TA. Psychotherapy for depression among incurable cancer patients. *Cochrane Database Syst Rev* 2008;2:CD005537.

18. Rodin G, Lloyd N, Katz M, Green E, Mackay JA, Wong RK. The treatment of depression in cancer patients: a systematic review. *Support Care Cancer* 2007;15(2):123-136.

19. Stiefel F, Die TM, Berney A, Olarte JM, Razavi A. Depression in palliative care: a pragmatic report from the Expert Working Group of the European Association for Palliative Care. *Support Care Cancer* 2001;9(7):477-488.

20. Williams S, Dale J. The effectiveness of treatment for depression/depressive symptoms in adults with cancer: a systematic review. *Br J Cancer* 2006;94(3):372-390.

21. Gothelf D, Rubinstein M, Shemesh E et al. Pilot study: fluvoxamine treatment for depression and anxiety disorders in children and adolescents with cancer. *J Am Acad Child Adolesc Psychiatry* 2005;44(12):1258-1262.

22. DeJong M, Fombonne E. Depression in paediatric cancer: an overview. *Psychooncology* 2006;15(7):553-566.

23. Fisch MJ, Loehrer PJ, Kristeller J et al. Fluoxetine versus placebo in advanced cancer outpatients: a double-blinded trial of the Hoosier Oncology Group. *J Clin Oncol* 2003;21(10):1937-1943.

24. Gill D, Hatcher S. Antidepressants for depression in medical illness. *Cochrane Database Syst Rev* 2000;4:CD001312.

25. Maisami M, Sohmer BH, Coyle JT. Combined use of tricyclic antidepressants and neuroleptics in the management of terminally ill children: a report on three cases. *J Am Acad Child Psychiatry* 1985;24(4):487-489.

26. Pfefferbaum-Levine B, Kumor K, Cangir A, Choroszy M, Roseberry EA. Tricyclic antidepressants for children with cancer. *Am J Psychiatry* 1983;140(8):1074-1076.

27. Walling VR, Pfefferbaum B. The use of methylphenidate in a depressed adolescent with AIDS. *J Dev Behav Pediatr* 1990;11(4):195-197.

28. Homsi J, Nelson KA, Sarhill N et al. A phase II study of methylphenidate for depression in advanced cancer. *Am J Hosp Palliat Care* 2001;18(6):403-407.

29. Rozans M, Dreisbach A, Lertora JJ, Kahn MJ. Palliative uses of methylphenidate in patients with cancer: a review. *J Clin Oncol* 2002;20(1):335-339.

30. Bausewein C, Booth S, Gysels M, Higginson I. Non-pharmacological interventions for breathlessness in advanced stages of malignant and non-malignant diseases. *Cochrane Database Syst Rev* 2008;2:CD005623.

31. Ben-Aharon I, Gafter-Gvili A, Paul M, Leibovici L, Stemmer SM. Interventions for alleviating cancer-related dyspnea: a systematic review. *J Clin Oncol* 2008;26(14):2396-2404.

32. Bredin M, Corner J, Krishnasamy M, Plant H, Bailey C, A'Hern R. Multicentre randomised controlled trial of nursing intervention for breathlessness in patients with lung cancer. *BMJ* 1999;318(7188):901-904.

33. Connors S, Graham S, Peel T. An evaluation of a physiotherapy led non-pharmacological breathlessness programme for patients with intrathoracic malignancy. *Palliat Med* 2007;21(4):285-287.

34. Corner J, Plant H, A'Hern R, Bailey C. Non-pharmacological intervention for breathlessness in lung cancer. *Palliat Med* 1996;10(4):299-305.

35. DiSalvo WM, Joyce MM, Tyson LB, Culkin AE, Mackay K. Putting evidence into practice: evidence-based interventions for cancer-related dyspnea. *Clin J Oncol Nurs* 2008;12(2):341-352.

36. Hately J, Laurence V, Scott A, Baker R, Thomas P. Breathlessness clinics within specialist palliative care settings can improve the quality of life and functional capacity of patients with lung cancer. *Palliat Med* 2003;17(5):410-417.

37. Booth S, Moosavi SH, Higginson IJ. The etiology and management of intractable breathlessness in patients with advanced cancer: a systematic review of pharmacological therapy. *Nat Clin Pract Oncol* 2008;5(2):90-100.

38. Freedman S. Facial cooling and perception of dyspnoea. *Lancet* 1987;2(8569):1215.

39. Schwartzstein RM, Lahive K, Pope A, Weinberger SE, Weiss JW. Cold facial stimulation reduces breathlessness induced in normal subjects. *Am Rev Respir Dis* 1987;136(1):58-61.

40. Mize WL. Clinical training in self-regulation and practical pediatric hypnosis: what pediatricians want pediatricians to know. *J Dev Behav Pediatr* 1996;17(5):317-322.

41. Ahmedzai S, Davis C. Nebulised drugs in palliative care. *Thorax* 1997;52 Suppl 2:S75-S77.

42. Jennings AL, Davies AN, Higgins JP, Gibbs JS, Broadley KE. A systematic review of the use of opioids in the management of dyspnoea. *Thorax* 2002;57(11):939-944.

43. Clemens KE, Quednau I, Klaschik E. Use of oxygen and opioids in the palliation of dyspnoea in hypoxic and non-hypoxic palliative care patients: a prospective study. *Support Care Cancer* 2009;17(4):367-377.

44. Viola R, Kiteley C, Lloyd NS, Mackay JA, Wilson J, Wong RK. The management of dyspnea in cancer patients: a systematic review. *Support Care Cancer* 2008;16(4):329-337.

45. Jennings AL, Davies AN, Higgins JP, Broadley K. Opioids for the palliation of breathlessness in terminal illness. *Cochrane Database Syst Rev* 2001;4:CD002066.

46. Polosa R, Blackburn MR. Adenosine receptors as targets for therapeutic intervention in asthma and chronic obstructive pulmonary disease. *Trends Pharmacol Sci* 2009;30(10):528-535.

47. Navigante AH, Cerchietti LC, Castro MA, Lutteral MA, Cabalar ME. Midazolam as adjunct therapy to morphine in the alleviation of severe dyspnea perception in patients with advanced cancer. *J Pain Symptom Manage* 2006;31(1):38-47.

48. Twycross R. The terminal phase. Oxford textbook of palliative medicine. Oxford University Press. Oxford,UK. 2008.

49. Booth S, Wade R, Johnson M, Kite S, Swannick M, Anderson H. The use of oxygen in the palliation of breathlessness. A report of the expert working group of the Scientific Committee of the Association of Palliative Medicine. *Respir Med* 2004;98(1):66-77.

50. Bruera E, Schoeller T, MacEachern T. Symptomatic benefit of supplemental oxygen in hypoxemic patients with terminal cancer: the use of the N of 1 randomized controlled trial. *J Pain Symptom Manage* 1992;7(6):365-368.

51. Cranston JM, Crockett AJ, Moss JR, Alpers JH. Domiciliary oxygen for chronic obstructive pulmonary disease. *Cochrane Database Syst Rev* 2005;4:CD001744.

52. Uronis HE, Currow DC, McCrory DC, Samsa GP, Abernethy AP. Oxygen for relief of dyspnoea in mildly- or non-hypoxaemic patients with cancer: a systematic review and meta-analysis. *Br J Cancer* 2008;98(2):294-299.

53. Beardsmore S, Fitzmaurice N. Palliative care in paediatric oncology. *Eur J Cancer* 2002;38(14):1900-1907.

54. Gleeson C, Spencer D. Blood transfusion and its benefits in palliative care. *Palliat Med* 1995;9(4):307-313.

55. Monti M, Castellani L, Berlusconi A, Cunietti E. Use of red blood cell transfusions in terminally ill cancer patients admitted to a palliative care unit. *J Pain Symptom Manage* 1996;12(1):18-22.

56. Buyukpamukcu M, Varan A, Kutluk T, Akyuz C. Is epoetin alfa a treatment option for chemotherapy-related anemia in children? *Med Pediatr Oncol* 2002;39(4):455-458.

57. Razzouk BI, Hord JD, Hockenberry M et al. Double-blind, placebo controlled study of quality of life, hematologic end points, and safety of weekly epoetin alfa in children with cancer receiving myelosuppressive chemotherapy. *J Clin Oncol* 2006;24(22):3583-3589.

58. Wilson J, Yao GL, Raftery J et al. A systematic review and economic evaluation of epoetin alpha, epoetin beta and darbepoetin alpha in anaemia associated with cancer, especially that attributable to cancer treatment. *Health Technol Assess* 2007;11(13):1-iv.

59. Seto AH, Dunlap DS. Tranexamic acid in oncology. *Ann Pharmacother* 1996;30(7-8):868-870.

60. Dean A, Tuffin P. Fibrinolytic inhibitors for cancer-associated bleeding problems. *J Pain Symptom Manage* 1997;13(1):20-24.

61. Brook L, Vickers J, Pizer B. Home platelet transfusion in pediatric oncology terminal care. *Med Pediatr Oncol* 2003;40(4):249-251.

62. Hirsh J, Siragusa S, Cosmi B, Ginsberg JS. Low molecular weight heparins (LMWH) in the treatment of patients with acute venous thromboembolism. *Thromb Haemost* 1995;74(1):360-363.

63. Crowther M, Hirsh J. Low-molecular-weight heparin for the out-of-hospital treatment of venous thrombosis: rationale and clinical results. *Semin Thromb Hemost* 1997;23(1):77-81.

64. Akl EA, Vasireddi SR, Gunukula S et al. Anticoagulation for the initial treatment of venous thromboembolism in patients with cancer. *Cochrane Database Syst Rev* 2011;6:CD006649.

65. Eddy NB, Friebel H, Hahn KJ, Halbach H. Codeine and its alternates for pain and cough relief . 4. Potential alternates for cough relief. *Bull World Health Organ* 1969;40(5):639-719.

66. Homsi J, Walsh D, Nelson KA. Important drugs for cough in advanced cancer. *Support Care Cancer* 2001;9(8):565-574.

67. Matthys H, Bleicher B, Bleicher U. Dextromethorphan and codeine: objective assessment of antitussive activity in patients with chronic cough. *J Int Med Res* 1983;11(2):92-100.

68. Homsi J, Walsh D, Nelson KA et al. A phase II study of hydrocodone for cough in advanced cancer. *Am J Hosp Palliat Care* 2002;19(1):49-56.

69. Luporini G, Barni S, Marchi E, Daffonchio L. Efficacy and safety of levodropropizine and dihydrocodeine on nonproductive cough in primary and metastatic lung cancer. *Eur Respir J* 1998;12(1):97-101.

70. Back IN, Jenkins K, Blower A, Beckhelling J. A study comparing hyoscine hydrobromide and glycopyrrolate in the treatment of death rattle. *Palliat Med* 2001;15(4):329-336.

71. Bennett M, Lucas V, Brennan M, Hughes A, O'Donnell V, Wee B. Using anti-muscarinic drugs in the management of death rattle: evidence-based guidelines for palliative care. *Palliat Med* 2002;16(5):369-374.

72. Clark K, Butler M. Noisy respiratory secretions at the end of life. *Curr Opin Support Palliat Care* 2009;3(2):120-124.

73. Hughes A, Wilcock A, Corcoran R, Lucas V, King A. Audit of three antimuscarinic drugs for managing retained secretions. *Palliat Med* 2000;14(3):221-222.

74. Wee B, Hillier R. Interventions for noisy breathing in patients near to death. *Cochrane Database Syst Rev* 2008;1:CD005177.

75. Wildiers H, Dhaenekint C, Demeulenaere P et al. Atropine, hyoscine butylbromide, or scopolamine are equally effective for the treatment of death rattle in terminal care. *J Pain Symptom Manage* 2009;38(1):124-133.

76. Wildiers H, Menten J. Death rattle: prevalence, prevention and treatment. *J Pain Symptom Manage* 2002;23(4):310-317.

77. Bosonnet L. Pruritus: scratching the surface. *Eur J Cancer Care (Engl )* 2003;12(2):162-165.

78. Evers AW, Casteelen G, Duller P. Multidisciplinaire diagnostiek en behandeling van complexe jeukproblematiek bij huidaandoeningen. *Nederlands tijdschrift voor dermatologie en venereologie* 2005;8:440-444.

79. Rucklidge JJ, Saunders D. The efficacy of hypnosis in the treatment of pruritus in people with HIV/AIDS: a time-series analysis. *Int J Clin Exp Hypn* 2002;50(2):149-169.

80. Rucklidge JJ, Saunders D. Hypnosis in a case of long-standing idiopathic itch. *Psychosom Med* 1999;61(3):355-358.

81. Sampson RN. Hypnotherapy in a case of pruritus and Guillain-Barre syndrome. *Am J Clin Hypn* 1990;32(3):168-173.

82. Ko MC, Song MS, Edwards T, Lee H, Naughton NN. The role of central mu opioid receptors in opioid-induced itch in primates. *J Pharmacol Exp Ther* 2004;310(1):169-176.

83. Maxwell LG, Kaufmann SC, Bitzer S et al. The effects of a small-dose naloxone infusion on opioid-induced side effects and analgesia in children and adolescents treated with intravenous patient-controlled analgesia: a double-blind, prospective, randomized, controlled study. *Anesth Analg* 2005;100(4):953-958.

84. Kjellberg F, Tramer MR. Pharmacological control of opioid-induced pruritus: a quantitative systematic review of randomized trials. *Eur J Anaesthesiol* 2001;18(6):346-357.

85. Borgeat A, Stirnemann HR. Ondansetron is effective to treat spinal or epidural morphine-induced pruritus. *Anesthesiology* 1999;90(2):432-436.

86. Charuluxananan S, Somboonviboon W, Kyokong O, Nimcharoendee K. Ondansetron for treatment of intrathecal morphine-induced pruritus after cesarean delivery. *Reg Anesth Pain Med* 2000;25(5):535-539.

87. Dimitriou V, Voyagis GS. Opioid-induced pruritus: repeated vs single dose ondansetron administration in preventing pruritus after intrathecal morphine. *Br J Anaesth* 1999;83(5):822-823.

88. Gurkan Y, Toker K. Prophylactic ondansetron reduces the incidence of intrathecal fentanyl-induced pruritus. *Anesth Analg* 2002;95(6):1763-6.

89. Kyriakides K, Hussain SK, Hobbs GJ. Management of opioid-induced pruritus: a role for 5-HT3 antagonists? *Br J Anaesth* 1999;82(3):439-441.

90. Aymard JP, Lederlin P, Witz F, Colomb JN, Herbeuval R, Weber B. Cimetidine for pruritus in Hodgkin's disease. *Br Med J* 1980;280(6208):151-152.

91. Harrison AR, Littenberg G, Goldstein L, Kaplowitz N. Pruritus, cimetidine, and polycythemia. *N Engl J Med* 1979;300(8):433-434.

92. Hess CE. Cimetidine for the treatment of pruritus. *N Engl J Med* 1979;300(7):370.

93. Schapira DV, Bennett JM. Cimetidine for pruritus. *Lancet* 1979;1(8118):726-727.

94. Staubli M, Graf W, Straub PW. [Pruritus in Hodgkin's disease responding to cimetidine]. *Schweiz Med Wochenschr* 1981;111(20):723-724.

95. Weick JK, Donovan PB, Najean Y et al. The use of cimetidine for the treatment of pruritus in polycythemia vera. *Arch Intern Med* 1982;142(2):241-242.

96. Zappacosta AR, Hauss D. Cimetidine doesn't help pruritus of uremia. *N Engl J Med* 1979;300(22):1280.

97. Korfitis C, Trafalis DT. Carbamazepine can be effective in alleviating tormenting pruritus in patients with hematologic malignancy. *J Pain Symptom Manage* 2008;35(6):571-572.

98. Zylicz Z, Smits C, Krajnik M. Paroxetine for pruritus in advanced cancer. *J Pain Symptom* Manage 1998;16(2):121-124.

99. Zylicz Z, Krajnik M, Sorge AA, Costantini M. Paroxetine in the treatment of severe non-dermatological pruritus: a randomized, controlled trial. *J Pain Symptom Manage* 2003;26(6):1105-1112.

100. Muller C, Pongratz S, Pidlich J et al. Treatment of pruritus in chronic liver disease with the 5-hydroxytryptamine receptor type 3 antagonist ondansetron: a randomized, placebo-controlled, double-blind cross-over trial. *Eur J Gastroenterol Hepatol* 1998;10(10):865-870.

101. O'Donohue JW, Pereira SP, Ashdown AC, Haigh CG, Wilkinson JR, Williams R. A controlled trial of ondansetron in the pruritus of cholestasis. *Aliment Pharmacol Ther* 2005;21(8):1041-1045.

102. Davis MP, Frandsen JL, Walsh D, Andresen S, Taylor S. Mirtazapine for pruritus. *J Pain Symptom Manage* 2003;25(3):288-291.

103. Demierre MF, Taverna J. Mirtazapine and gabapentin for reducing pruritus in cutaneous T-cell lymphoma. *J Am Acad Dermatol* 2006;55(3):543-544.

104. Hundley JL, Yosipovitch G. Mirtazapine for reducing nocturnal itch in patients with chronic pruritus: a pilot study. *J Am Acad Dermatol* 2004;50(6):889-891.

105. Browning J, Combes B, Mayo MJ. Long-term efficacy of sertraline as a treatment for cholestatic pruritus in patients with primary biliary cirrhosis. *Am J Gastroenterol* 2003;98(12):2736-2741.

106. Diehn F, Tefferi A. Pruritus in polycythaemia vera: prevalence, laboratory correlates and management. *Br J Haematol* 2001;115(3):619-621.

107. Mayo MJ, Handem I, Saldana S, Jacobe H, Getachew Y, Rush AJ. Sertraline as a first-line treatment for cholestatic pruritus. *Hepatology* 2007;45(3):666-674.

108. Stander S, Bockenholt B, Schurmeyer-Horst F et al. Treatment of chronic pruritus with the selective serotonin re-uptake inhibitors paroxetine and fluvoxamine: results of an open-labelled, two-arm proof-of-concept study. *Acta Derm Venereol 2009*;89(1):45-51.

109. Tefferi A, Fonseca R. Selective serotonin reuptake inhibitors are effective in the treatment of polycythemia vera-associated pruritus. *Blood* 2002;99(7):2627.

110. Datta DV, Sherlock S. Cholestyramine for long term relief of the pruritus complicating intrahepatic cholestasis. *Gastroenterology* 1966;50(3):323-332.

111. Di PC, Tritapepe R, Rovagnati P, Rossetti S. Double-blind placebo-controlled clinical trial of microporous cholestyramine in the treatment of intra- and extra-hepatic cholestasis: relationship between itching and serum bile acids. *Methods Find Exp Clin Pharmacol* 1984;6(12):773-776.

112. Tandon P, Rowe BH, Vandermeer B, Bain VG. The efficacy and safety of bile Acid binding agents, opioid antagonists, or rifampin in the treatment of cholestasis-associated pruritus. *Am J Gastroenterol* 2007;102(7):1528-1536.

113. Balistreri WF. Bile acid therapy in pediatric hepatobiliary disease: the role of ursodeoxycholic acid. *J Pediatr Gastroenterol Nutr* 1997;24(5):573-589.

114. Dinler G, Kocak N, Yuce A, Gurakan F, Ozen H. Ursodeoxycholic acid therapy in children with cholestatic liver disease. *Turk J Pediatr* 1999;41(1):91-98.

115. El-Karaksy H, Mansour S, El-Sayed R, El-Raziky M, El-Koofy N, Taha G. Safety and efficacy of rifampicin in children with cholestatic pruritus. *Indian J Pediatr* 2007;74(3):279-281.

116. Khurana S, Singh P. Rifampin is safe for treatment of pruritus due to chronic cholestasis: a meta-analysis of prospective randomized-controlled trials. *Liver Int* 2006;26(8):943-948.

117. Ghent CN, Bloomer JR, Hsia YE. Efficacy and safety of long-term phenobarbital therapy of familial cholestasis. *J Pediatr* 1978;93(1):127-132.

118. Cies JJ, Giamalis JN. Treatment of cholestatic pruritus in children. *Am J Health Syst Pharm* 2007;64(11):1157-1162.

119. Chang Y, Golkar L. The use of naltrexone in the management of severe generalized pruritus in biliary atresia: report of a case. *Pediatr Dermatol* 2008;25(3):403-404.

120. Bergasa NV, Talbot TL, Alling DW et al. A controlled trial of naloxone infusions for the pruritus of chronic cholestasis. *Gastroenterology* 1992;102(2):544-549.

121. Bergasa NV, Alling DW, Talbot TL et al. Effects of naloxone infusions in patients with the pruritus of cholestasis. A double-blind, randomized, controlled trial. *Ann Intern Med* 1995;123(3):161-167.

122. Connolly CS, Kantor GR, Menduke H. Hepatobiliary pruritus: what are effective treatments? *J Am Acad Dermatol* 1995;33(5 Pt 1):801-805.

123. Jones EA, Neuberger J, Bergasa NV. Opiate antagonist therapy for the pruritus of cholestasis: the avoidance of opioid withdrawal-like reactions. *QJM* 2002;95(8):547-552.

124. V&VN Oncologie. Oral mucositis. National Guideline. Oncoline Version 1.0. <http://www.oncoline.nl/orale-mucositis>. 2007.

125. Zeltzer L, LeBaron S, Zeltzer PM. The effectiveness of behavioral intervention for reduction of nausea and vomiting in children and adolescents receiving chemotherapy. *J Clin Oncol* 1984;2(6):683-690.

126. LeBaron S, Zeltzer L. Behavioral intervention for reducing chemotherapy-related nausea and vomiting in adolescents with cancer. *J Adolesc Health Care* 1984;5(3):178-182.

127. Cotanch P, Hockenberry M, Herman. Self-hypnosis as antiemetic therapy in children receiving chemotherapy. *Oncol Nurs Forum* 1985;12(4):41-46.

128. Hockenberry MJ, Cotanch PH. Hypnosis as adjuvant antiemetic therapy in childhood cancer. *Nurs Clin North Am* 1985;20(1):105-107.

129. Jacknow DS, Tschann JM, Link MP, Boyce WT. Hypnosis in the prevention of chemotherapy-related nausea and vomiting in children: a prospective study. *J Dev Behav Pediatr* 1994;15(4):258-264.

130. Ahles TA, Tope DM, Pinkson B et al. Massage therapy for patients undergoing autologous bone marrow transplantation. *J Pain Symptom Manage* 1999;18(3):157-163.

131. Cassileth BR, Vickers AJ. Massage therapy for symptom control: outcome study at a major cancer center. *J Pain Symptom Manage* 2004;28(3):244-249.

132. Grealish L, Lomasney A, Whiteman B. Foot massage. A nursing intervention to modify the distressing symptoms of pain and nausea in patients hospitalized with cancer. *Cancer Nurs* 2000;23(3):237-243.

133. Vasterling J, Jenkins RA, Tope DM, Burish TG. Cognitive distraction and relaxation training for the control of side effects due to cancer chemotherapy. *J Behav Med* 1993;16(1):65-80.

134. Luebbert K, Dahme B, Hasenbring M. The effectiveness of relaxation training in reducing treatment-related symptoms and improving emotional adjustment in acute non-surgical cancer treatment: a meta-analytical review. *Psychooncology* 2001;10(6):490-502.

135. Devine EC, Westlake SK. The effects of psychoeducational care provided to adults with cancer: meta-analysis of 116 studies. *Oncol Nurs Forum* 1995;22(9):1369-1381.

136. Ezzone S, Baker C, Rosselet R, Terepka E. Music as an adjunct to antiemetic therapy. *Oncol Nurs Forum* 1998;25(9):1551-1556.

137. Vickers AJ. Can acupuncture have specific effects on health? A systematic review of acupuncture antiemesis trials*. J R Soc Med* 1996;89(6):303-311.

138. Ezzo JM, Richardson MA, Vickers A et al. Acupuncture-point stimulation for chemotherapy-induced nausea or vomiting. *Cochrane Database Syst Rev* 2006;2:CD002285.

139. Naeim A, Dy SM, Lorenz KA, Sanati H, Walling A, Asch SM. Evidence-based recommendations for cancer nausea and vomiting. *J Clin Oncol* 2008;26(23):3903-3910.

140. Brown S, North D, Marvel MK, Fons R. Acupressure wrist bands to relieve nausea and vomiting in hospice patients: do they work? *Am J Hosp Palliat Care* 1992;9(4):26-29.

141. Nystrom E, Ridderstrom G, Leffler AS. Manual acupuncture as an adjunctive treatment of nausea in patients with cancer in palliative care--a prospective, observational pilot study. *Acupunct Med* 2008;26(1):27-32.

142. Perkins P, Vowler SL. Does acupressure help reduce nausea and vomiting in palliative care patients? Pilot study. *Palliat Med* 2008;22(2):193-194.

143. Wright LD. The use of motion sickness bands to control nausea and vomiting in a group of hospice patients. *Am J Hosp Palliat Care* 2005;22(1):49-53.

144. Uysal KM, Olgun N, Sarialioglu F. Tropisetron in the prevention of chemotherapy-induced acute emesis in pediatric patients. *Turk J Pediatr* 1999;41(2):207-218.

145. Ozkan A, Yildiz I, Yuksel L, Apak H, Celkan T. Tropisetron (Navoban) in the control of nausea and vomiting induced by combined cancer chemotherapy in children. *Jpn J Clin Oncol* 1999;29(2):92-95.

146. Aksoylar S, Akman SA, Ozgenc F, Kansoy S. Comparison of tropisetron and granisetron in the control of nausea and vomiting in children receiving combined cancer chemotherapy. *Pediatr Hematol Oncol* 2001;18(6):397-406.

147. Herrstedt J, Roila F. Chemotherapy-induced nausea and vomiting: ESMO clinical recommendations for prophylaxis. *Ann Oncol* 2008;19 Suppl 2:ii110-ii112.

148. Kris MG, Hesketh PJ, Somerfield MR et al. American Society of Clinical Oncology guideline for antiemetics in oncology: update 2006. *J Clin Oncol* 2006;24(18):2932-2947.

149. Roila F, Hesketh PJ, Herrstedt J. Prevention of chemotherapy- and radiotherapy-induced emesis: results of the 2004 Perugia International Antiemetic Consensus Conference. *Ann Oncol* 2006;17(1):20-28.

150. Santucci G, Mack JW. Common gastrointestinal symptoms in pediatric palliative care: nausea, vomiting, constipation, anorexia, cachexia. *Pediatr Clin North Am* 2007;54(5):673-89.

151. Ventaffrida V, Oliveri E, Caraceni A et al. A retrospective study on the use of oral morphine in cancer pain. *J Pain Symptom Manage* 1987;2(2):77-81.

152. Drake R, Longworth J, Collins JJ. Opioid rotation in children with cancer. *J Palliat Med* 2004;7(3):419-422.

153. Buchanan D, Muirhead K. Intractable nausea and vomiting successfully related with granisetron 5-hydroxytryptamine type 3 receptor antagonists in Palliative Medicine. *Palliat Med* 2007;21(8):725-726.

154. Cole RM, Robinson F, Harvey L, Trethowan K, Murdoch V. Successful control of intractable nausea and vomiting requiring combined ondansetron and haloperidol in a patient with advanced cancer. *J Pain Symptom Manage* 1994;9(1):48-50.

155. Currow DC, Coughlan M, Fardell B, Cooney NJ. Use of ondansetron in palliative medicine. *J Pain Symptom Manage* 1997;13(5):302-307.

156. Ljutic D, Perkovic D, Rumboldt Z, Bagatin J, Hozo I, Pivac N. Comparison of ondansetron with metoclopramide in the symptomatic relief of uremia induced nausea and vomiting. *Kidney Blood Press Res* 2002;25(1):61-64.

157. Mystakidou K, Befon S, Trifyllis J, Liossi C, Papadimitriou J. Tropisetron versus Metoclopramide in the Control of Emesis in Far-Advanced Cancer. *Oncologist* 1997;2(5):319-323.

158. Mystakidou K, Befon S, Liossi C, Vlachos L. Comparison of the efficacy and safety of tropisetron, metoclopramide, and chlorpromazine in the treatment of emesis associated with far advanced cancer. *Cancer* 1998;83(6):1214-1223.

159. Nicholson S, Evans C, Mansi J. Ondansetron in intractable nausea and vomiting. *Lancet* 1992;339(8791):490.

160. Porcel JM, Salud A, Porta J, Schoenenberger JA. Antiemetic efficacy of subcutaneous 5-HT3 receptor antagonists in terminal cancer patients. *J Pain Symptom Manage* 1998;15(5):265-266.

161. Sussman G, Shurman J, Creed MR et al. Intravenous ondansetron for the control of opioid-induced nausea and vomiting. International S3AA3013 Study Group. *Clin Ther* 1999;21(7):1216-1227.

162. Critchley P, Plach N, Grantham M et al. Efficacy of haloperidol in the treatment of nausea and vomiting in the palliative patient: a systematic review. *J Pain Symptom Manage* 2001;22(2):631-634.

163. Perkins P, Dorman S. Haloperidol for the treatment of nausea and vomiting in palliative care patients. *Cochrane Database Syst Rev* 2009;2:CD006271.

164. Eisenchlas JH, Garrigue N, Junin M, De Simone GG. Low-dose levomepromazine in refractory emesis in advanced cancer patients: an open-label study. *Palliat Med* 2005;19(1):71-75.

165. Kennett A, Hardy J, Shah S, A'Hern R. An open study of methotrimeprazine in the management of nausea and vomiting in patients with advanced cancer. *Support Care Cancer* 2005;13(9):715-721.

166. Skinner J, Skinner A. Levomepromazine for nausea and vomiting in advanced cancer. *Hosp Med* 1999;60(8):568-570.

167. Smith AR, Repka TL, Weigel BJ. Aprepitant for the control of chemotherapy induced nausea and vomiting in adolescents. *Pediatr Blood Cancer* 2005;45(6):857-860.

168. Diemunsch P, Gan TJ, Philip BK et al. Single-dose aprepitant vs ondansetron for the prevention of postoperative nausea and vomiting: a randomized, double-blind phase III trial in patients undergoing open abdominal surgery. *Br J Anaesth* 2007;99(2):202-211.

169. Gore L, Chawla S, Petrilli A et al. Aprepitant in adolescent patients for prevention of chemotherapy-induced nausea and vomiting: a randomized, double-blind, placebo-controlled study of efficacy and tolerability. *Pediatr Blood Cancer* 2009;52(2):242-247.

170. Hall W, Degenhardt L. Medical marijuana initiatives : are they justified? How successful are they likely to be? *CNS Drugs* 2003;17(10):689-697.

171. Bruera E, Seifert L, Watanabe S et al. Chronic nausea in advanced cancer patients: a retrospective assessment of a metoclopramide-based antiemetic regimen. *J Pain Symptom Manage* 1996;11(3):147-153.

172. Bruera E, Moyano JR, Sala R et al. Dexamethasone in addition to metoclopramide for chronic nausea in patients with advanced cancer: a randomized controlled trial. *J Pain Symptom Manage* 2004;28(4):381-388.

173. Hardy JR, Rees E, Ling J et al. A prospective survey of the use of dexamethasone on a palliative care unit. *Palliat Med* 2001;15(1):3-8.

174. Mystakidou K, Befon S, Liossi C, Vlachos L. Comparison of tropisetron and chlorpromazine combinations in the control of nausea and vomiting of patients with advanced cancer. *J Pain Symptom Manage* 1998;15(3):176-184.

175. Riad W, Altaf R, Abdulla A, Oudan H. Effect of midazolam, dexamethasone and their combination on the prevention of nausea and vomiting following strabismus repair in children. *Eur J Anaesthesiol* 2007;24(8):697-701.

176. Davis MP, Hallerberg G. A systematic review of the treatment of nausea and/or vomiting in cancer unrelated to chemotherapy or radiation. *J Pain Symptom Manage* 2010;39(4):756-767.

177. Kearsley JH, Williams AM, Fiumara AM. Antiemetic superiority of lorazepam over oxazepam and methylprednisolone as premedicants for patients receiving cisplatin-containing chemotherapy. *Cancer* 1989;64(8):1595-1599.

178. Bishop JF, Olver IN, Wolf MM et al. Lorazepam: a randomized, double-blind, crossover study of a new antiemetic in patients receiving cytotoxic chemotherapy and prochlorperazine. *J Clin Oncol* 1984;2(6):691-695.

179. Lundstrom S, Zachrisson U, Furst CJ. When nothing helps: propofol as sedative and antiemetic in palliative cancer care. *J Pain Symptom Manage* 2005;30(6):570-577.

180. Glover ML, Kodish E, Reed MD. Continuous propofol infusion for the relief of treatment-resistant discomfort in a terminally ill pediatric patient with cancer. *J Pediatr Hematol Oncol* 1996;18(4):377-380.

181. Gan TJ, Ginsberg B, Grant AP, Glass PS. Double-blind, randomized comparison of ondansetron and intraoperative propofol to prevent postoperative nausea and vomiting. *Anesthesiology* 1996;85(5):1036-1042.

182. Borgeat A, Wilder-Smith OH, Saiah M, Rifat K. Subhypnotic doses of propofol possess direct antiemetic properties. *Anesth Analg* 1992;74(4):539-541.

183. Ndao DH, Ladas EJ, Cheng B et al. Inhalation aromatherapy in children and adolescents undergoing stem cell infusion: results of a placebo-controlled double-blind trial. *Psychooncology* 2012;21(3):247-254.

184. Vlieger AM, Menko-Frankenhuis C, Wolfkamp SC, Tromp E, Benninga MA. Hypnotherapy for children with functional abdominal pain or irritable bowel syndrome: a randomized controlled trial. *Gastroenterology* 2007;133(5):1430-1436.

185. Eccleston C, Palermo TM, Williams AC, Lewandowski A, Morley S. Psychological therapies for the management of chronic and recurrent pain in children and adolescents. *Cochrane Database Syst Rev* 2009;2:CD003968.

186. World Health Organization. WHO guidelines on the pharmalogical treatment of persisting pain in children with medical illnesses. [http://whqlibdoc.who.int/publications/2012/9789241548120_Guidelines.pdf](http://whqlibdoc.who.int/publications/2012/9789241548120_Guidelines.pdf     %0D%09%09%092012)

[2012](http://whqlibdoc.who.int/publications/2012/9789241548120_Guidelines.pdf     %0D%09%09%092012).

187. Wiffen PJ, Edwards JE, Barden J, McQuay HJ. Oral morphine for cancer pain. *Cochrane Database Syst Rev* 2003;4:CD003868.

188. Saarto T, Wiffen PJ. Antidepressants for neuropathic pain. *Cochrane Database Syst Rev* 2007;4:CD005454.

189. Finnerup NB, Sindrup SH, Jensen TS. The evidence for pharmacological treatment of neuropathic pain. *Pain* 2010;150(3):573-581.

190. Moore RA, Straube S, Wiffen PJ, Derry S, McQuay HJ. Pregabalin for acute and chronic pain in adults. *Cochrane Database Syst Rev* 2009;3:CD007076.

191. Moore RA, Wiffen PJ, Derry S, McQuay HJ. Gabapentin for chronic neuropathic pain and fibromyalgia in adults. *Cochrane Database Syst Rev* 2011;3:CD007938.

192. Eisenberg E, McNicol E, Carr DB. Opioids for neuropathic pain. *Cochrane Database Syst Rev* 2006;3:CD006146.

193. Mercadante S, Ferrera P, Villari P, David F, Giarratano A, Riina S. Effects of red blood cell transfusion on anemia-related symptoms in patients with cancer. *J Palliat Med* 2009;12(1):60-63.

194. Davies B, Whitsett SF, Bruce A, McCarthy P. A typology of fatigue in children with cancer. *J Pediatr Oncol Nurs* 2002;19(1):12-21.

195. Radbruch L, Strasser F, Elsner F et al. Fatigue in palliative care patients -- an EAPC approach. *Palliat Med* 2008;22(1):13-32.

196. National Comprehensive Cancer Network. NCCN Clinical Practice Guideline Cancer related fatigue. http://www.nccn.org/professionals/physician_gls/f_guidelines.asp.2013.

197. Mock V, Atkinson A, Barsevick AM et al. Cancer-related fatigue. Clinical Practice Guidelines in Oncology. *J Natl Compr Canc Netw* 2007;5(10):1054-1078.

198. Cramp F, Daniel J. Exercise for the management of cancer-related fatigue in adults. *Cochrane Database Syst Rev* 2008;2:CD006145.

199. Headley JA, Ownby KK, John LD. The effect of seated exercise on fatigue and quality of life in women with advanced breast cancer. *Oncol Nurs Forum* 2004;31(5):977-983.

200. Kangas M, Bovbjerg DH, Montgomery GH. Cancer-related fatigue: a systematic and meta-analytic review of non-pharmacological therapies for cancer patients. *Psychol Bull* 2008;134(5):700-741.

201. Oldervoll LM, Loge JH, Paltiel H et al. The effect of a physical exercise program in palliative care: A phase II study. *J Pain Symptom Manage* 2006;31(5):421-430.

202. Porock D, Kristjanson LJ, Tinnelly K, Duke T, Blight J. An exercise intervention for advanced cancer patients experiencing fatigue: a pilot study. *J Palliat Care* 2000;16(3):30-36.

203. Stricker CT, Drake D, Hoyer KA, Mock V. Evidence-based practice for fatigue management in adults with cancer: exercise as an intervention. *Oncol Nurs Forum* 2004;31(5):963-976.

204. Temel JS, Greer JA, Goldberg S et al. A structured exercise program for patients with advanced non-small cell lung cancer. *J Thorac Oncol* 2009;4(5):595-601.

205. Brunnhuber K. Putting evidence into practice: palliative care. <https://www.unitedhealthfoundation.org/uhfassets/docs/2008/ebm-brunnhuber-nash-meier-weissman-woodcock-palliative.pdf>. BMJ Publishing Group. 2008.

206. Armes J, Chalder T, Addington-Hall J, Richardson A, Hotopf M. A randomized controlled trial to evaluate the effectiveness of a brief, behaviorally oriented intervention for cancer-related fatigue. *Cancer* 2007;110(6):1385-1395.

207. Barsevick AM, Dudley W, Beck S, Sweeney C, Whitmer K, Nail L. A randomized clinical trial of energy conservation for patients with cancer-related fatigue. *Cancer* 2004;100(6):1302-1310.

208. Ream E, Richardson A, Alexander-Dann C. Supportive intervention for fatigue in patients undergoing chemotherapy: a randomized controlled trial. *J Pain Symptom Manage* 2006;31(2):148-161.

209. Auret KA, Schug SA, Bremner AP, Bulsara M. A randomized, double-blind, placebo-controlled trial assessing the impact of dexamphetamine on fatigue in patients with advanced cancer. *J Pain Symptom Manage* 2009;37(4):613-621.

210. Breitbart W, Rosenfeld B, Kaim M, Funesti-Esch J. A randomized, double- blind, placebo-controlled trial of psychostimulants for the treatment of fatigue in ambulatory patients with human immunodeficiency virus disease. *Arch Intern Med* 2001;161(3):411-420.

211. Bruera E, Valero V, Driver L et al. Patient-controlled methylphenidate for cancer fatigue: a double-blind, randomized, placebo-controlled trial. *J Clin Oncol* 2006;24(13):2073-2078.

212. Minton O, Richardson A, Sharpe M, Hotopf M, Stone P. A systematic review and meta-analysis of the pharmacological treatment of cancer-related fatigue. *J Natl Cancer Inst* 2008;100(16):1155-1166.
